# Instrumental activities of daily living and self-rated health in community-dwelling older adults: cross-sectional findings from the HUNT Study (HUNT4 Trondheim 70+)

**DOI:** 10.1186/s12877-025-05983-w

**Published:** 2025-05-14

**Authors:** Anne Kusk Pedersen, Linda Ernstsen

**Affiliations:** 1 Department of Public Health and Nursing, Norwegian University of Science and Technology, Postbox 8905, N-7491, Trondheim, Norway; 2 Bispebjerg Hospital, Department of Neurology, Copenhagen, Denmark

**Keywords:** Instrumental Activities of Daily Living (IADL), Self-rated health (SRH), Cognitive function, Community-dwelling older people, Epidemiology

## Abstract

**Background:**

The population of older adults is growing, posing new challenges for society and healthcare services. Instrumental Activities of Daily Living (IADL) describe individuals’ ability to handle more complex activities in their daily life and to the extent to which they can live independently. Self-rated health (SRH) is a frequently used metric in health research and is a robust predictor for institutionalization and mortality. Therefore, the purpose of this study is to investigate the association between IADL function and SRH among community-dwelling older adults in Norway, and to determine the influence of cognitive function.

**Methods:**

A total of 1104 community-dwelling adults aged 70 or older participating in the population-based Trøndelag Health Study (HUNT) 4 Trondheim 70 + were included. Logistic regression was used to examine the association between IADL function and SRH. IADL function was divided into two groups, IADL limitation (requiring help to complete one or more daily activities) and no IADL limitation. SRH were dichotomized into good and poor SRH.

**Results:**

Of the 1104 included participants 127 (11.5%) experienced IADL limitation. After adjustment for gender, age, cohabiting status, educational level, physical activity, gait speed, grip strength, depressive symptoms, limiting long-term illness and cognitive function, participants with IADL limitation had significantly higher odds of poor SRH compared to those without IADL limitation (odds ratio 3.26, 95% confidence interval 1.89–5.61, *p* < 0.001).

**Conclusions:**

These cross-sectional results from an urban population of community-dwelling older adults showed a strong association between IADL limitation and poor SRH independent of cognitive function, emphasizing the importance of investigating the prospective relationship between IADL and SRH. Intervention studies are needed to confirm whether improving IADL function can impact SRH in older adults.

**Clinical trial number:**

Not applicable.

## Background

The global population of older adults is on the rise, and by 2050 it is estimated that nearly 22% of the world’s population will be aged 60 or older and almost double the current proportion of 12%. Additionally, the numbers of individuals aged 80 years and over is projected to triple between 2020 and 2050 [1]. This demographic shift brings with it a higher prevalence of chronic diseases and disorders, including musculoskeletal conditions, cancer, cardiovascular diseases, and dementia.

Despite evidence suggesting that later-born birth cohorts have better health [2], the aging population will inevitably lead to an increased demand for healthcare services and support for older adults [3]. Activities of Daily Living (ADL) is a concept developed to assess the functional level of older adults [4]. It encompasses both personal (PADL) and instrumental (IADL) activities. IADL activities are more complex than PADL and require higher cognitive functioning [4, 5]. IADL is crucial for older adults to live independently, but limitations in IADL increase with age for both men and women, with women experiencing greater limitations than men [6, 7]. Other studies indicate that low social participation [8–10], low level of physical activity [8, 11, 12], physical function measured through grip strength [13, 14] and gait speed [14], depressive symptoms [8, 11, 15], and cognitive function [8, 9, 15] seem to predict IADL limitation. In turn, dependency in older adults has shown to be associated with morbidity and mortality [16], and that mental and cognitive disorders independently increase the risk of care dependence [17].

Self-rated health (SRH) is a commonly used measure of individuals’ health [18]. It encompasses social, psychological, and biological factors [19]. It is observed to be a strong predictor of institutionalization [20, 21] and mortality [18, 22]. Some researchers have found that SRH is a stronger predictor of mortality among men than women [22], while others find no difference [18], or find it to be stronger among women compared to men [23]. A recent population-based study from Norway showed that among older adults over 70 years more women report poorer SRH than men [24]. However, it`s worth noting that more women participated in the study, and their mean age was significantly higher compared to men. Despite women having a longer life-expectancy than men in most countries, their disability-free expectancy is falling behind, according to findings from the United States [25]. Most studies have examined the association between SRH and IADL, but a few studies suggest an association between IADL and SRH [26–28]. Cognitive function and dementia significantly impact IADLs, making the assessment of these abilities crucial for diagnosing dementia and managing care plans. However, the predictive value of cognition on IADL limitations [8, 9, 15] may have led to the notion that IADL limitations are related to mild cognitive impairment and dementia. Therefore, it is of great importance to assess whether the association between IADL and SRH in community-dwelling older adults is affected by cognitive function. The aim of this study is to examine the association between IADL function and SRH among community-dwelling older adults, and to assess the influence of cognitive function.

## Methods

### Study sample

**Fig. 1:**
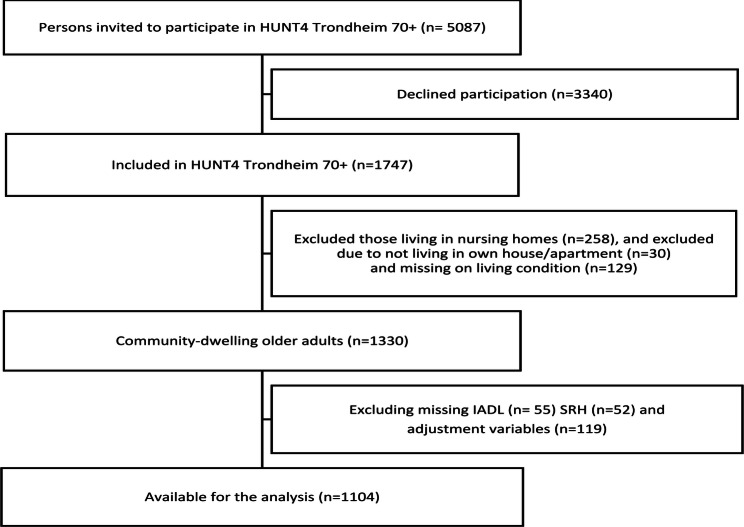
Flowchart for the study sample from HUNT4 Trondheim 70+

In the fourth wave of the population based Trøndelag Health Study (HUNT), carried out in the Trøndelag county (2018-19), a substudy with focus on older adults’ health (HUNT4 70+) was conducted [29]. The HUNT4 70 + survey expanded its original catchment area to include parts of Trondheim city, aiming to incorporate an urban population. As of 2023, Trondheim had a population of 212,660 and was the third most populous municipality and the fourth largest urban area in Norway [30]. For the current cross-sectional study, we use data from an urban population of community-dwelling older adults participating in the HUNT4 Trondheim 70 + study. The assessors (health personnel and nursing students) received a standardized two-day training in dementia assessment and other measurement before data collection began. A field station was set up where participants underwent health examinations, standardized interviews and questionnaires. For those who requested it, the assessments were conducted at home. Physical performance was assessed with the Short Physical Performance Battery (SPPB) [31]. All participants, whether assessed at the field stations or at home, were evaluated using the same protocol [32]. Assessment of dementia consisted of questions and tests relating to cognition, daily life functioning, neuropsychiatric symptoms, subjective cognitive impairment, possible onset of symptoms and development of dementia symptoms in line with the protocol used in the larger HUNT4 70 + study [33]. The Montreal Cognitive Assessment (MoCA) test was used to test cognition [34]. A diagnostic work-up of medical doctors with experience in research and clinical work in geriatrics, old-age psychiatry and neurology group made diagnoses of dementia and mild cognitive impairment (MCI) for each individual, using the DSM-5 criteria [33]. In total 5087 older adults were invited, and 1747 participated (a participation rate of 34.3%) [32], see Fig. 1. We excluded those living in nursing homes (*n* = 258) or not living in own house/apartment (*n* = 30), and those who had missing data for cohabiting status (*n* = 129). Additionally, participants with any missing values on IADL (*n* = 55), SRH (*n* = 52), and the adjustment variables (*n* = 119) were excluded. The final study sample consisted of *n* = 1104 participants (shown in Fig. 1).

### Study variables

#### Independent variable

The independent variable *IADL* function was defined using the question “Can you, without help from others, do the following tasks?” and the answer options were ‘yes’ or ‘no’ for each activity. IADL included 8 activities from the HUNT questionnaire, based on the validated instrument ‘Lawton & Brody Instrumental Activity of Daily Living Scale’ [35]. These activities encompassed preparing warm meals, performing light housework, engaging in heavier housework, doing the laundry, shopping, managing bill payments, administering prescribed medicines, and taking the bus. IADL limitation was defined as needing help from another person in one or more IADL activities (yes/no).

#### Dependent variable

The outcome variable *SRH* was assessed using the question “How is your present state of health?”. The participants could choose from the following options: ‘very good’, ‘good’, ‘fair’ and ‘poor’. We further dichotomized this variable into good (combining very good and good) and poor (combining fair and poor) SRH.

#### Adjustment variables

*Gender* was registered at participation in two categories: women and men. *Age* in years was used as continuous variable. *Cohabiting status* was dichotomized into living alone or living with a spouse or others aged 18 or older. *Educational level* was divided in three groups: low, medium and high. Those with low level of education had completed primary school (≤ 10 years). Medium educational level refers to completed secondary school or an apprenticeship (11–13 years). Participants with high level of education had completed less or more than four years of tertiary education (in total ≥ 14 years) and were used as reference. *Physical activity* was based on frequency of exercise and categorized into four groups: never or less than once a week, once a week, 2–3 times a week, about every day. *Gait speed* (meter/second) was measured twice over a 4-m distance at the participants preferred/normal pace, using the faster of the two values. Gait speed was divided into quartiles, and highest quartile was used as reference in the analysis. *Grip strength* was measured in kilogram (kg) by using the Jamar Plus + Digital Hand Dynamometer^®^ (Performance Health, Warrenwille, USA). Three trials were performed consecutively without resting pauses for each hand with the person in sitting position, the arm hanging free by the body, the shoulder adducted and neutrally rotated, the elbow held in 90° and the wrist in neutral position. The instruction was to squeeze as hard as possible. Dominant hand was registered. We used the best measurement in the analyses. Due to established gender differences in grip strength raw maximum grip strength in kilogram (kg) is presented separately for women and men in the descriptive table. For the regression analyses these values are converted to a gender adjusted z-score (mean subtracted and divided by the standard deviation (SD)), based on the gender specific values in the total study sample. *Depressive symptoms* were measured using the validated Hospital Anxiety and Depression Scale (HADS) [36, 37]. According to depressive symptoms, seven questions are included. Total score has a range from 0 to 21, and a score ≥ 8 indicates considerable depressive symptoms and possible depression [38]. Missing values on one or two of the seven questions was replaced with a mean score made from the answered questions. The variable was used as a dichotomous variable with a cut-off on 8. *Limiting long-term illness* was measured with the question ‘Do you have any long-term illnesses (lasting at least 1 year), injuries or disorders of a physical or mental nature that impair your function level in daily life?’. Participants could answer ‘yes’ or ‘no’ and the variable consisted of the two groups. *Cognitive function* was used as a continuous variable, measured with the validated screening tool The Montreal Cognitive Assessment (MoCA) [34]. It is possible to get a score between 0 and 30, and the higher the score indicates the better cognitive function. In the adjusted analyses MoCA was used as a continuous variable. More information about the cognitive assessment in HUNT4 Trondheim 70 + can be found in the article by Gjøra et al. [33].

### Statistical analysis

Descriptive statistics were used to present the characteristics of the study participants according to IADL function. Binary logistic regression was employed to describe the association between IADL limitation and poor SRH. The association is presented using odds ratios (OR) with 95% confidence intervals (95% CI) in 4 models. First, an unadjusted analysis was conducted (model 1). In model 2, adjustments were made for gender, age, cohabiting status, and educational level. In model 3, adjustments were additionally made for model 2 and physical activity, gait speed and grip strength. In model 4, adjustments were made for model 3 and depressive symptoms, limiting long-term illness, and cognitive function. The analysis suggested no statistical interaction (*p* = 0.746) between IADL limitations and gender, and there were overall few IADL limitations in the study sample, thus stratified analyses by gender were not conducted. Additionally, a sensitivity analysis was performed to examine whether the relationship between IADL and SRH changed significantly when participants with dementia were excluded. All analysis were performed using IBM SPSS Statistics. P values of < 0.0.5 (two-tailed) were considered statistically significant. Microsoft Copilot, an artificial intelligence tool, was used for proofreading and improving the language of this manuscript.

### Ethics approval and consent to participate

Participation in the HUNT4 Trondheim 70 + study was based on informed consent. For persons with reduced capacity to consent, written consent was obtained from the next of kin. The current study was approved by the Regional Committee for Medical and Health Research Ethics Central Norway (REC Central Norway 85430). The study was conducted in accordance with the principles of the Declaration of Helsinki, ensuring that all research involving human participants was carried out with integrity, transparency, and respect for human rights, safety, and well-being.

**Results**.

In this study we included 1104 participants with an average age of 76.3 years (± 5.06), age range 70.1–95.9 years. In total 597 of the participants were women (54.1%). In the total sample 127 (11.5%) participants had IADL limitation, 977 (88.5%) had no IADL limitation. In the group with IADL limitation 50.4% of the participants reported poor SRH. In the group with no IADL limitation only 11.6% of the participants reported poor SRH. For the entire study sample 16% reported poor SRH. Additionally, 68 (6.2%) participants had dementia and 390 (35.3%) had MCI.

Table 1 presents the participants by IADL limitation (yes/no). Notably, the average age was 5.8 years higher for the group with IADL limitation compared to the group without IADL limitation (81.5 years vs. 75.7 years). Furthermore, a larger proportion of those with IADL limitation lived alone (42.5% vs. 29.7%), and a greater percentage had a low educational level (22% vs. 7.5%).

**Table 1:** Descriptive statistics of participants with and without IADL limitation at baseline (*n* = 1104)

Variables	IADL limitation (*n* = 127)	No IADL limitation (*n* = 977)
**Poor self-rated health**	64 (50.4)	113 (11.6)
**Gender**, women	71 (55.9)	526 (53.8)
**Age** in years, mean (SD)	81.5 (6.6)	75.7 (4.4)
**Living alone**	54 (42.5)	290 (29.7)
**Educational level**		
High Medium Low	54 (42.5) 45 (35.4) 28 (22)	530 (54.2) 374 (38.3) 73 (7.5)
**Physical activity**		
About every day 2–3 times a week Once a week Never or less than once a week	26 (20.5) 46 (36.2) 17 (13.4) 38 (29.9)	392 (40.1) 449 (46.0) 83 (8.5) 53 (5.4)
**Gait speed (meter/second)**		
Score, mean (SD)	0.77 (0.24)	1.06 (0.23)
Quartiles		
Q1 (1.19+)	5 (3.9)	261 (26.7)
Q2 (1.19–1.03)	9 (7.1)	273 (27.9)
Q3 (1.03–0.87) Q4 (< 0.87)	28 (22.0) 85 (66.9)	251 (25.7) 192 (19.7)
**Grip strength (kilogram)**,** women**		
Score, mean (SD)	21.95 (5.72)	25.70 (4.90)
**Grip strength (kilogram)**,** men**		
Score, mean (SD)	34.13 (8.92)	42.25 (7.22)
**Depressive symptoms**		
HADS-score^c^, mean (SD) HADS ≥8	4.4 (2.9) 20 (15.7)	2.6 (2.3) 42 (4.3)
**Limiting long-term illness**	102 (80.3)	407 (41.7)
**Cognitive function**		
MoCA-score^d^, mean (SD) No cognitive impairment Mild cognitive impairment Dementia	21.9 (4.8) 61 (48.0) 37 (29.1) 29 (22.8)	24.4 (3.2) 585 (59.9) 353 (36.1) 39 (4.0)

IADL; Instrumental Activities of Daily Living. HUNT; The Trøndelag Health Study. Data are presented as numbers and percentage (%) and as mean and standard deviation (SD). Educational level, high = less or more than four years of tertiary education, medium = secondary school or an apprenticeship, low = primary school. Physical activity was divided into quartiles: never or less than once a week, once a week, 2–3 times a week, about every day. Gait speed were divided intro quartiles, Q1, Q2, Q3 and Q4. Grip strength as gender adjusted z-score. Depressive symptoms are defined as score ≥8 on ^c^Hospital Anxiety and Depression Scale (HADS). Cognitive function assessed with ^d^Montreal Cognitive Assessment (MoCA)-score

In the group with IADL limitation, the proportion of individuals who were active never or less than once a week was considerable higher compared to those without IADL-limitation (29.9% vs. 5.4%). The same applied to physical function measured as proportion within the lowest quartiles of gait speed (66.9% vs. 19.7%) and measured as grip strength in kilograms for both women (21.95 (± 5.72) vs. 25.70 (± 4.90)) and men (34.13 (± 8.92) vs. 42.25 (± 7.22)). Among participants with IADL limitation, 15.7% exhibited depressive symptoms with a HADS score of ≥8, whereas only 4.3% of participants without IADL limitation had similar symptoms. Additionally, 80.3% of participants with IADL limitation reported having a chronic illness in contrast to 41.7% in the group without IADL limitation. The average MoCA score for the group with IADL limitation was 22.9 (± 4.8), while it was 24.4 (± 3.2) for the group without IADL limitation. In the group with IADL limitation, 22.8% of the participants had received a diagnosis of dementia, whereas in the group without IADL limitation, only 4.0% of the participants had received this diagnosis. Most participants with IADL limitation faced challenges in performing heavier housework (7.3%), followed by taking the bus (4.3%) and doing laundry (3.8%) (as shown in Table 2).

**Table 2:** The prevalence of IADL limitations by activity in the sample (*n* = 1104)

IADL limitation	*n*	(%)
Prepare warm meals	14	(1.3)
Do light housework	10	(0.9)
Do heavier housework	81	(7.3)
Do the laundry	42	(3.8)
Do the shopping	23	(2.1)
Pay bills	30	(2.7)
Take the medicines	14	(1.2)
Take the bus	48	(4.3)
No IADL limitation	977	(88.5)
Total	1104	(100)

**Fig. 2:**
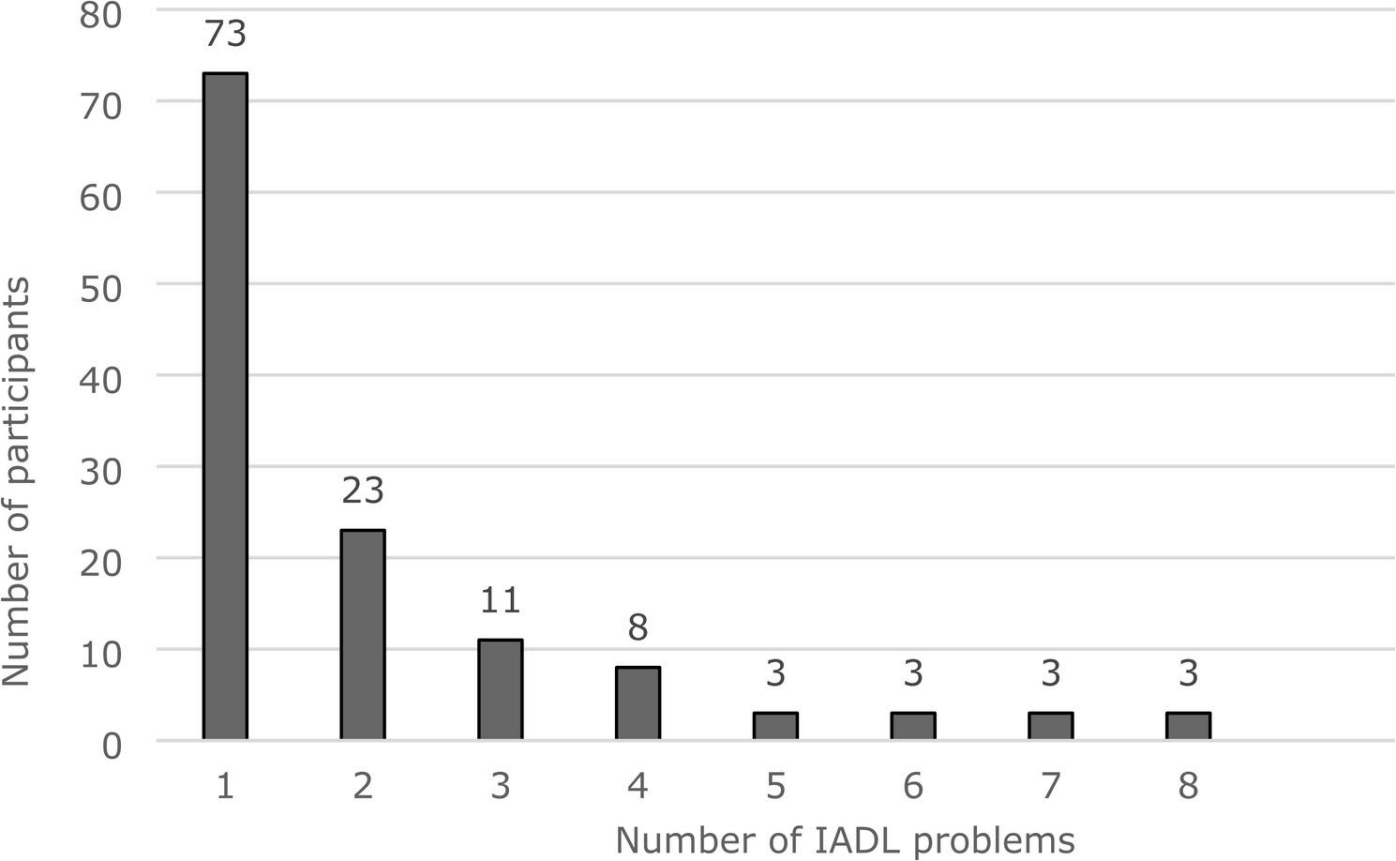
The distribution of IADL limitations among those reporting one or more challenges (*n* = 127)

Of the 127 participants who had IADL limitation, 73 participants experienced limitation with performing one of the 8 activities. Only three of the participants in this group needed assistance with all 8 activities: preparing warm meals, performing light housework, engaging in heavier housework, doing the laundry, shopping, managing bill payments, administering prescribed medicines, and taking the bus (shown in Fig. 2).

The results from the logistic regression analysis revealed a significant association between IADL limitation and poor SRH (as shown in Table 3). The inclusion of depressive symptoms, limiting long-term illness and cognitive functioning in model 4 substantially reduced the association between IADL and SRH. Still, the results in the fully adjusted model 4 showed that the odds for reporting poor SRH was 3.26 times higher (95% CI 1.89–5.61) for participants with IADL limitation compared to participants without IADL limitation.

**Table 3:** Association between IADL limitation and poor SRH (*n*=1104). Binary logistic regression, method = enter. OR (95% CI)

	Model 1		Model 2		Model 3		Model 4	
	OR	95% CI	OR	95% CI	OR	95% CI	OR	95% CI
No IADL limitation	1.00	Reference	1.00	Reference	1.00	Reference	1.00	Reference
IADL limitation	7.77	(5.21–11.58)	7.29	(4.63–11.47)	4.87	(2.98–7.96)	3.26	(1.89–5.61)

**Model 1**: Unadjusted

**Model 2**: Adjusted for gender, age, cohabiting status and educational level

**Model 3**: Adjusted for model 2 + physical activity, gait speed and grip strength

**Model 4**: Adjusted for model 3 + depressive symptoms, limiting long-term illness and cognitive function

In the sensitivity analyses, excluding those with a dementia diagnosis (*n* = 68), the OR for poor SRH in individuals with IADL limitation in the fully adjusted model (model 4) was 3.31 (95% CI 1.84–5.94). This in comparison to an OR of 3.26 (95% CI 1.89–5.61) in the fully adjusted model that included those with dementia. Our finding demonstrates that the association between IADL limitation and poor SRH was not strongly influenced by the inclusion of participants with dementia; therefore, they were included in our study sample.

## Discussion

The aim of this study was to investigate the relationship between IADL function and SRH among community-dwelling older Norwegians. We found that 127 (11.5%) participants had IADL limitation. In the group with IADL limitation, 50.4% reported poor SRH, while for the entire sample 14.6% reported poor SRH. Participants with IADL limitation were older, a larger proportion lived alone, and their educational level was lower compared to participants without IADL limitation. The results from the fully adjusted analysis showed that those having IADL limitation had significantly higher odds of reporting poor SRH compared to participants without IADL limitation.

The prevalence of IADL limitation was lower in this study compared to studies among older adults from America [39], Asia [26] and Europe [7, 16], including older Norwegian adults in HUNT3 (2006-08) [11]. Additionally, recent numbers from the European Health Interview Survey (EHIS) found IADL-limitations in Norwegian adults 70 years or older to be 30%, which is a substantial higher than 11.5% in our study sample [40]. However, the questions used to assess IADL limitations in EHIS differ from those used in HUNT4 Trondheim 70+. It is also plausible that older adults with multiple IADL limitations are likely less inclined to participate in population-based studies involving clinical measurements compared to telephone interviews or postal surveys. Thus, it is crucial to acknowledge that methodological differences in defining and assessing the IADL variable may limit direct comparison of IADL prevalence across studies. When comparing the different study populations, there are differences in educational and income level. Although income data was not accessible for this study, the proportion with a higher level of education (52.9%) was significantly larger than the national average among Norwegian adults aged 67 years or older [41]. Both educational and income level are factors tend to exhibit an inverse association with the prevalence of IADL limitation [42, 43]. In general, educational level in Norway is higher in the largest cities, and a recent paper using data from Norwegian birth cohorts (1965–1989) found that urban-rural differences in higher education have become more pronounced for recent cohorts, especially post-1980, and are more evident for men than women [44]. This highlights the need for further research on spatial inequalities in IADL among older women and men.

We found an association between having IADL limitation and reporting poor SRH, which is supported by previous research [26–28]. In the study by Byun et al. [26] older participants with IADL limitation had 1.80 times higher odds (95% CI: 1.52–2.13) of reporting poor SRH. However, in this study a modified assessment tool with cultural adaptions to measure IADL limitation was used, thus these findings cannot be compared directly with our results. Other studies have found associations for each of the IADL activities to be associated with SRH [27, 28]. Hu et al. [27] reported that disabilities with transport outside and responsibility for administering own medication were significantly associated with poor SRH. Gama et al. [28] found a strong association between independence in IADL activities (such as ability to handle finances, laundry, transport) and good SRH. These findings are partly supported from our result as the three most prevalent IADL disabilities in the study sample were heavier household, followed by taking the bus and doing the laundry. However, due to the low level of participants reporting challenges with several IADL categories we were not able to run separate analyses for the association between each IADL category and SRH.

In our study we did not find support for gender differences in the association between IADL limitation and poor SRH. Women and men had the same mean age as well as prevalence of IADL limitation. This despite of studies showing differences among older men and women according to IADL limitation, with women experiencing IADL limitation more frequently than men [6, 27]. However as pointed out by Allen et al. [45], in some cases, men’s need for assistance with IADL activities may not stem from functional impairment, but rather from their perception that such tasks are traditionally ‘women’s work’. Previously, activities such as cooking, housework, and laundry were excluded when scoring the IADL level for men, as these activities were not necessarily considered representative of men [35]. Today, these activities are treated equally, and all 8 activities are used for both genders [46].

In our study sample, we included participants with MCI (*n* = 390) and those with a dementia diagnosis (*n* = 68). However, in the fully adjusted model including adjustment for global cognitive function we still found a strong, statistically significant association between IADL and SRH. Sensitivity analysis indicated that excluding participants diagnosed with dementia did not substantially alter the result. While IADL is commonly used in dementia mapping, and that many studies show an association between cognitive function and IADL function [5, 47, 48], our findings emphasize that older adults with IADL difficulties that older adults with difficulties in IADL function should not be automatically associated with cognitive impairment or potential dementia. Evaluation of IADL remains important regardless of cognitive function.

### **Strength and limitations**

The strength of this study is that we used validated instruments to measure the independent variable IADL and the dependent variable SRH with data from the HUNT4 Trondheim 70 + study, providing valuable insight into older adult’s health.

There are several weaknesses in this study. Firstly, in HUNT4 Trondheim 70 + there was a low response rate, which is a limitation for internal validity. There is no available information about those who did not participate in HUNT4 Trondheim 70+, but in the HUNT4 study, non-participants reported poorer SRH, a higher prevalence of diseases, and a larger proportion had a dementia diagnosis compared to those who participated [29]. It is likely that the same tendencies are present in HUNT4 Trondheim 70+, resulting in a relatively lower proportion of participants with IADL limitation compared to the general population, indicating a healthy user bias. Secondly, we found that women and men 70–85 years in our study had higher mean grip strength assessed as kilograms (kg) with less standard errors (SEs) compared to the same age group in a representative sample of Norwegian adults [49]. The women and men had a mean grip strength of 25.3 kg (0.2) and 41.4 kg (0.3), respectively compared to women 24.3 kg (0.4) and men 40.0 kg (0.9) in the Norwegian Kan3 study (2022-23) [49]. These latter findings in addition to the high proportion of participants with higher educational level in our study sample compared to national average [41], suggest that the results may not be generalizable to all community-dwelling older Norwegians over 70 years of age. Thirdly, the IADL function is self-reported. This means that the IADL function does not necessarily reflects what the older adults managed in their daily lives but rather how they perceived their ability to perform these activities. This also implies that the assessment is at risk of both over- and underestimation. Fourthly, the dichotomization of the IADL will not capture the variance in the need of help of performing IADL activities. One should be aware of the bidirectional association, where many studies find that SRH affects IADL function [11, 50, 51]. As our study has a cross-sectional design causality between IADL function cannot be established, meaning that prospective studies on the interrelationship between IADL functions and SRH are needed. Variables we did not have access to could also have influenced the results, for example income level and prescribed drug use.

## Conclusion

In this sample of relatively healthy, community-dwelling older adults from an urban population we found a strong association between IADL function and SRH, which could not be solely explained by cognitive function. As the proportion of older adults rises, future population-based studies should investigate whether the development of IADL limitation in adulthood is associated with incident poor SRH in older age, further, if prevalence of IADL-limitations differs between older adults in urban versus rural settings. Additionally, intervention studies are needed to confirm whether improvements in IADL limitations can enhance SRH in community-dwelling older adults. Recognizing the link between everyday activities and SRH is crucial for designing more effective, personalized care and prevention plans for this population.

## Data Availability

The data supporting the findings of this study are available from the HUNT database. However, due to licensing restrictions, these data are not publicly accessible. Data may be obtained from the authors upon reasonable request, pending approval from the HUNT database.

## Abbreviations

IADL: Instrumental Activities of Daily Living
SRH: Self-rated health
HUNT: The Trøndelag Health Study
PADL: Personal Activities of Daily Living HADS: Hospital Anxiety and Depression Scale
MoCA: Montreal Cognitive Assessment
MCI: Mild cognitive impairment
OR: Odds ratio
SD: Standard deviation
